# ST-Elevation Myocardial Infarction (STEMI) in a Patient with Moyamoya Disease

**DOI:** 10.1155/2019/7124072

**Published:** 2019-02-04

**Authors:** James Livesay, Jeffrey Johnson

**Affiliations:** Department of Medicine, University of Tennessee Graduate School of Medicine, Knoxville, TN, USA

## Abstract

Moyamoya disease is a rare condition that is primarily reported in Asian populations, characterized by stenoocclusive intracranial angiopathy with small, fragile, and multiple collateral vessel formation. Extracranial complications, mainly abnormalities within the renal vasculature, have been described; however, there are very few case reports of cardiovascular complications in patients with Moyamoya disease. We report a 26-year-old Caucasian female with known Moyamoya disease who presented with both typical and atypical chest pain, mimicking symptoms of a previous non-ST-elevation myocardial infarction. Approximately six months prior to the current hospital admission, she underwent coronary angiography requiring percutaneous coronary intervention (PCI) with two drug-eluting stents to the right coronary artery (RCA) for a critical stenosis. Despite medical management, our patient developed inferior lead ST-elevations leading to a repeat left heart catheterization which showed clinically significant stenosis of the first obtuse marginal branch. Development of significant coronary artery stenosis in a short period of time demonstrates the clinical significance of minimal atherosclerosis in the setting of underlying fibrocellular thickening as seen in patients with Moyamoya disease. Clinicians need to be aware of the possibility of coronary involvement in addition to intracranial vascular complications in patients with Moyamoya disease and take appropriate measures to prevent or delay the development of atherosclerosis in these arteries.

## 1. Introduction

Moyamoya disease is a rare condition, primarily reported in Japanese and other Asian populations, best described by stenoocclusive changes in the internal carotid arteries and developed collateral vasculature [1]. These patients typically present at a young age with intracranial features such as ischemic or hemorrhagic strokes, and the occurrence of extracranial vascular dysfunction is exceptionally rare [2]. Reports of extracranial dysfunction in a patient with Moyamoya disease have mostly involved the renal vasculature leading to chronic kidney disease, while coronary artery involvement is extremely uncommon. The pathology of Moyamoya disease involves fibrocellular thickening of the vessel wall, and in this patient population, even minimal atherosclerosis can rapidly lead to significant stenosis of the coronary arteries [2]. Our patient, with known Moyamoya disease, presented with recurrent chest pain and stenosis in several coronary arteries that was successfully treated with multiple drug-eluting stents.

## 2. Case Presentation

A 26-year-old Caucasian female with known Moyamoya disease presented with a chief complaint of right-sided chest pain and numbness radiating to her right arm mimicking symptoms of a previous non-ST-elevation myocardial infarction.

**Figure 1:**
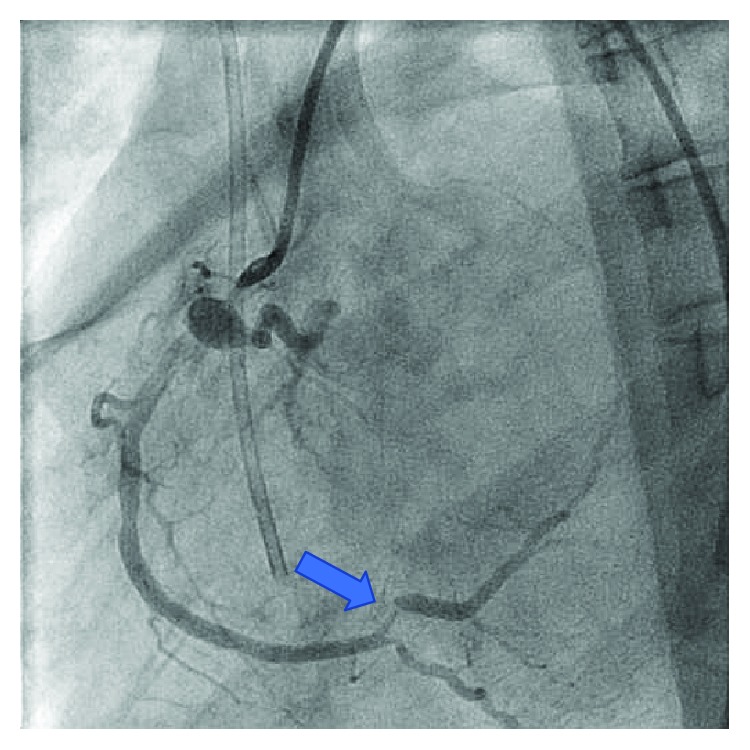
Previous critical RCA stenosis before stent placement.

**Figure 2:**
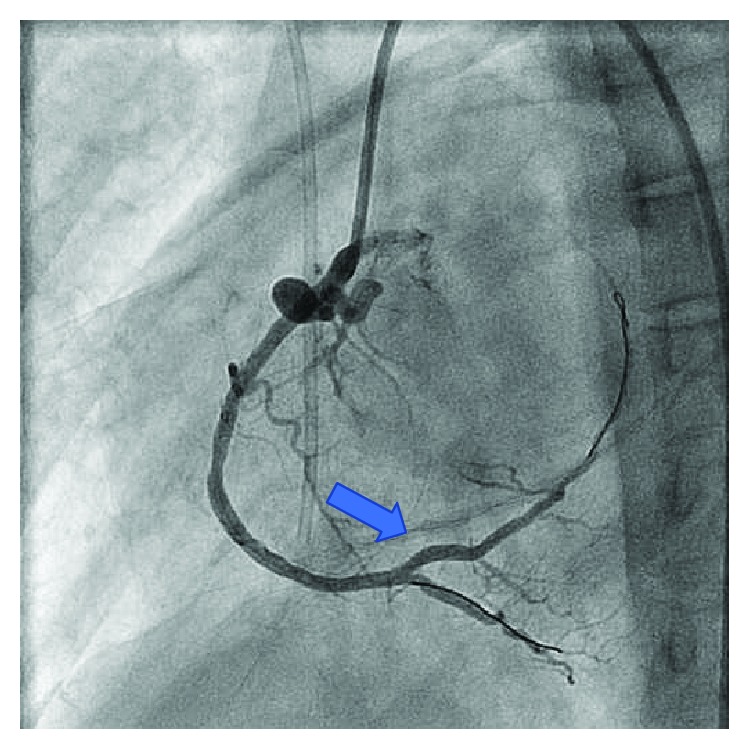
Previous critical RCA stenosis after stent placement.

Six months prior, the patient underwent coronary angiography remarkable for a critical lesion of the right distal coronary artery (Figure 1). At that time, she underwent successful percutaneous intervention with two drug-eluting stents to the distal right coronary artery (Figure 2). She had multiple other comorbidities including coronary artery disease, restrictive cardiomyopathy, hyperlipidemia, chronic kidney disease stage IV, anemia of chronic disease, recurrent pneumonia, immunoglobulin deficiency, osteoporosis, and history of cerebral vascular disease. In the months leading up to this admission, she suffered multiple noncardiac complications including symptomatic anemia, pneumonia with hemoptysis, and lower extremity ulcerations. She closely followed with hematology requiring Procrit for hemoglobin levels less than 7.0 g/dL with notable improvement in fatigue and weakness. She was also referred to dermatology for suspicious lower extremity lesions that were biopsied and felt to be autoimmune with intentions on starting IVIG.

Upon this admission, her initial troponin was 0.12 ng/mL which continued to rise to a maximum of 0.79 ng/mL over the first 24 hours; however, no specific EKG changes were noted. Given her rising troponin, presenting symptoms, and recently diagnosed coronary artery disease, she was started on guideline-directed medical therapy for acute coronary syndrome including heparin and beta blockade with as needed nitroglycerin, which provided significant improvement in symptoms. Due to the initial resolution of symptoms with medical management and associated high-morbidity conditions, we deferred left heart catheterization. Other significant initial labs included a 62 mg/dL BUN, 4.52 mg/dL creatinine, 23.0 mEq/L anion gap, 9.4 g/dL hemoglobin, 29.6% hematocrit, 18.2 white blood cell count, and 394 platelet count. A bedside echocardiogram was preformed revealing a normal left ventricular size with moderately reduced function with an LVEF of 40-45% along with mild global hypokinesis.

Over the subsequent 72 hours, she continued to have intermittent angina and her cardiac biomarkers began to climb, but serial EKGs did not display any signs of ST-segment changes. During this time, we obtained daily complete blood cell counts revealing a declining hemoglobin, and over time, a fall in her blood pressure was noted. Ultimately, we made a diagnosis of a type II non-ST-elevation myocardial infarction, believed to be secondary to demand ischemia. Therefore, we began blood transfusions to maintain a hemoglobin greater than 8.0 g/dL providing a resolution of anginal type symptoms.

**Figure 3:**
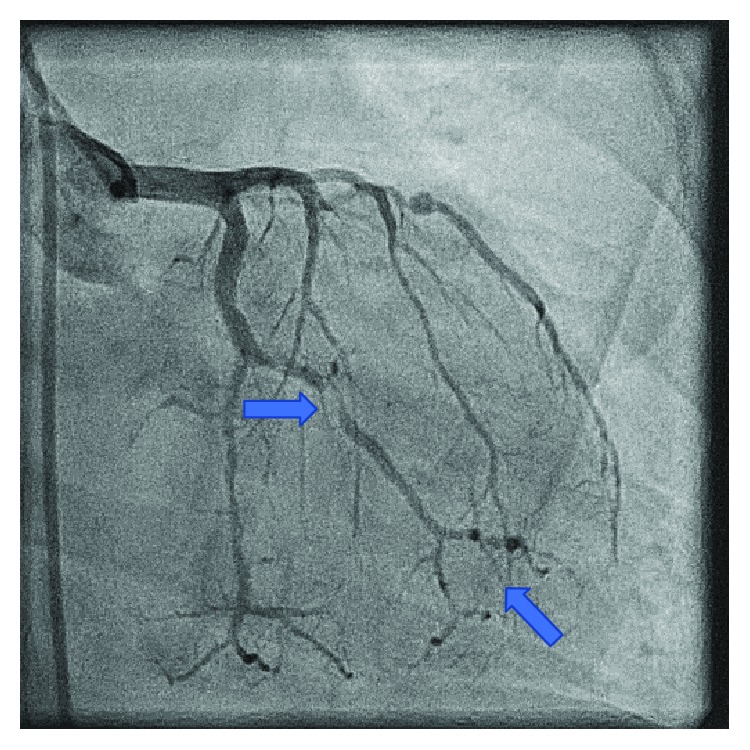
Stenosis of the obtuse marginal branch before stent placement. Distal obtuse marginal shows a string of bead appearance.

**Figure 4:**
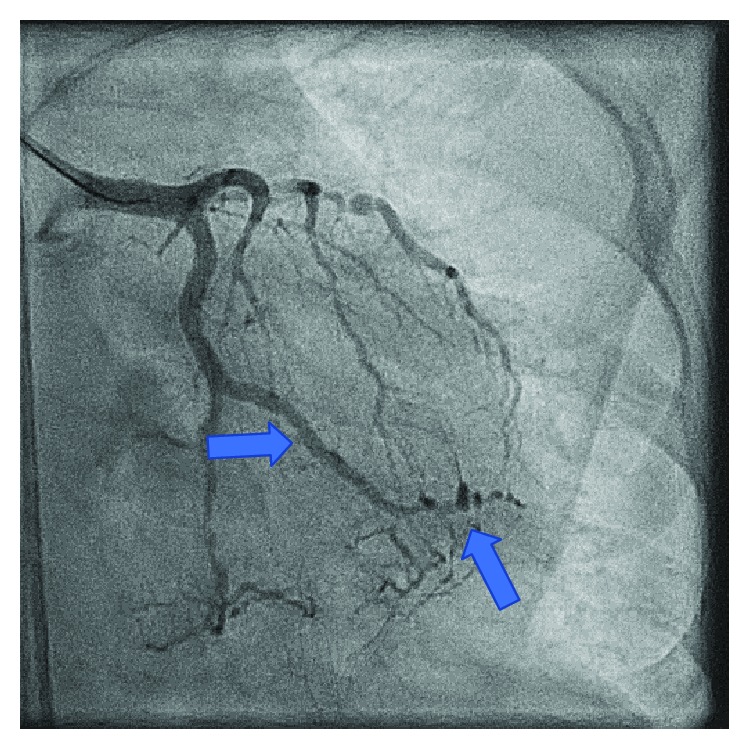
Stenosis of the obtuse marginal branch after stent placement. Distal obtuse marginal shows a string of bead appearance.

**Figure 5:**
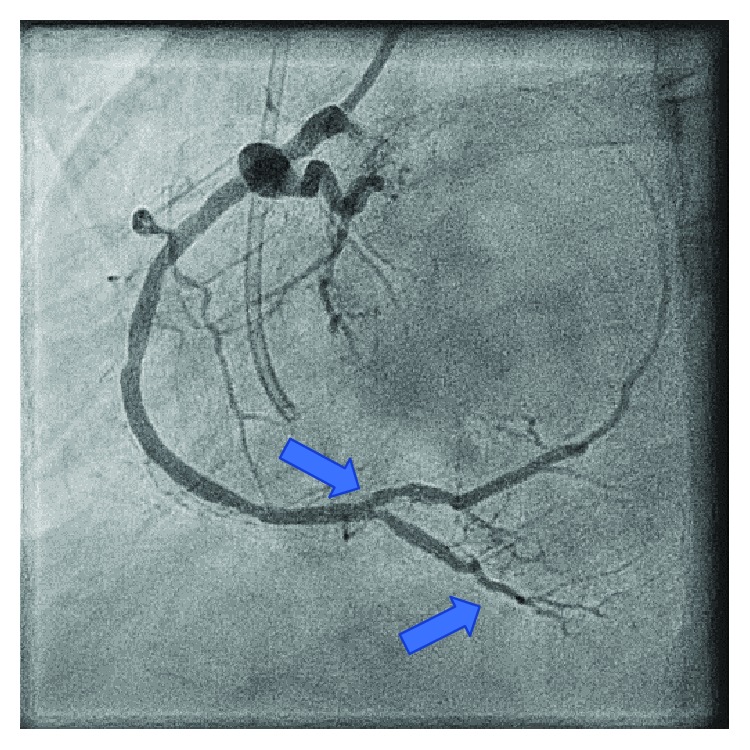
Restenosis of a previously placed RCA stent. Distal RCA shows a string of bead appearance.

**Figure 6:**
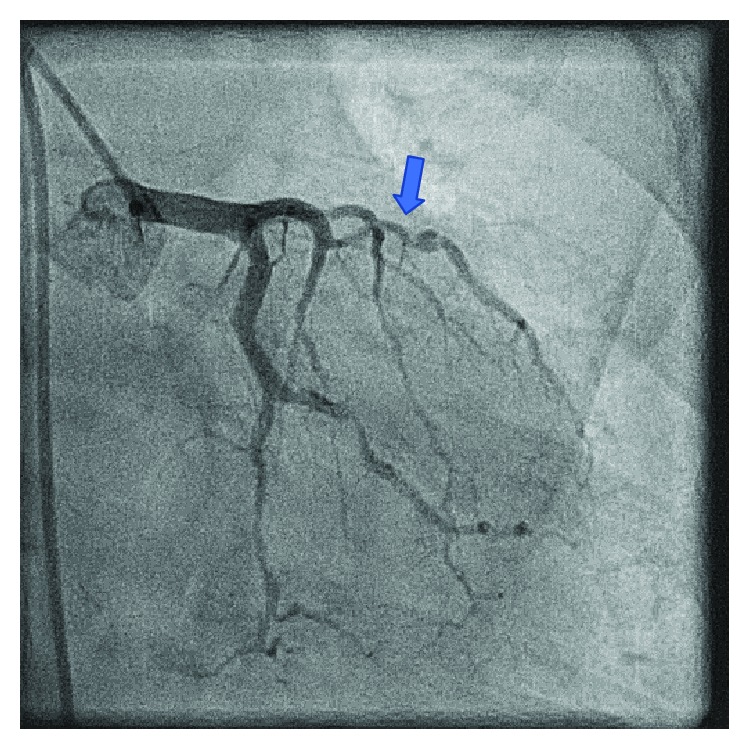
Stenosis of the first diagonal branch demonstrating multivessel involvement.

Despite continued medical management and improvement in her hemoglobin level, our patient again developed persistent recurrent angina. On hospital day nine, she developed angina not relieved by nitroglycerin, and a repeat EKG revealed inferior lead ST-elevations consistent with acute ST-elevation myocardial infarction. She underwent left heart catheterization remarkable for 90% stenosis of the first obtuse marginal branch (Figure 3), which was revascularized with a third drug-eluting stent (Figure 4). Diagnostic catheterization also revealed new 40-50% restenosis of her previous distal RCA stent (Figure 5) and noncritical stenosis of the first diagonal branch (Figure 6).

During this prolonged hospitalization, our patient suffered further complications including superficial femoral artery ischemia requiring intervention, acute on chronic renal failure, and sepsis secondary to pneumonia. Due to her worsening overall condition and multiple comorbidities, our patient elected to go home with hospice and ultimately passed away.

## 3. Discussion

Moyamoya disease is a rare disease that occurs in the Asian population, most often clinically manifested as an ischemic or hemorrhagic stroke at a young age. Cases of coronary artery disease secondary to Moyamoya disease are exceedingly rare with one article noting only 12 documented cases [3].

Choi and colleagues describe a patient with known Moyamoya disease that presented with recurrent chest pain aggravated by stress and relieved by nitroglycerin. This patient was diagnosed with variant angina after receiving two doses of ergonovine revealing 95% stenosis of the proximal LAD along with fibrous plaque occupying 33% of the coronary lumen [2]. A case reported by Komiyama and colleagues demonstrates a 56-year-old female that presented with angina with secondary to clinically significant LAD disease treated initially with balloon angioplasty followed by CABG several months later for restenosis [4].

Fibrocellular thickening of the intima and proliferative smooth muscle cell growth are the primary causes of arterial occlusion in Moyamoya disease. Nam and colleagues performed a retrospective study and postulated that symptomatic coronary heart disease is most likely the result of small atherosclerosis due to underlying endothelial proliferation [1]. Another study used intravascular ultrasound (IVUS) and virtual histology (VH) to evaluate a critical proximal LAD lesion in a 20-year-old Korean male with known Moyamoya disease. IVUS and VH revealed a homogenous, eccentric, echogenic intimal thickening composed of fibrous tissue with no mid or distal intimal thickening. Stenotic lesions seen in individuals with Moyamoya disease are distinctly different when compared to lesions seen in patients with CAD from atherosclerosis as there is minimal lipid pooling and no calcium deposition [5].

Our patient's coronary angiography showed several areas where the vessel resembles a string of beads, which is best seen in the distal OM branch and distal RCA (Figures 3, 4, and 5). The string of bead appearance has been previously described in other vessels such as the peripheral pulmonary, renal, and carotid arteries as a direct result of intimal thickening [6]. This distinct pattern helps clinicians distinguish CAD in Moyamoya disease from CAD in the setting of atherosclerosis using coronary angiography.

A recent study, using genetic analysis, found an association with homozygosity for *RNF213* p.Arg4810Lys and multiple extracranial vasculopathies. The p.Arg4810Lys variant, commonly found in the Eastern Asian population, is thought to cause the classic intracranial vasculopathy when in the heterozygous state. However, individuals that possess the homozygous variant seem to develop systemic vasculopathy and more severe symptoms [6]. The link between the RNF variant, p.Arg4810Lys, and Moyamoya disease has potential to be a screening tool for the risk of systemic disease and disease severity.

To the best of our knowledge, there are no other documented cases of coronary artery disease in a Caucasian female with Moyamoya disease. In this case, we were able to carefully track our patient's CAD progression from the time of her original NSTEMI, secondary to a critical RCA lesion, to her STEMI only six months later. Repeat coronary angiography revealed an unexpected critical lesion in a completely different vessel, the first distal obtuse marginal artery, along with mild RCA restenosis.

Regarding our patient's disease progression, she was originally diagnosed with Moyamoya disease at a young age after a typical presentation of a cerebral vascular accident requiring coiling by interventional radiology. Her disease continued to advance to include chronic renal failure, peripheral vascular disease, and finally coronary artery disease. As demonstrated from previous studies, even minimal atherosclerosis can lead to critical vascular lesions in a Moyamoya patient [1]. Therefore, we believe patients with the diagnosis of Moyamoya disease, especially with any extracranial features, should be counseled on their risk of heart disease. Clinicians following patients with Moyamoya disease should consider aggressive medical management to prevent plaque progression as minimal atherosclerosis can drastically increase this patient population's morbidity and mortality.

## 4. Conclusion

Extracranial vascular complications in patients with Moyamoya disease are rare but can have a significant impact on a patient's overall morbidity and mortality. Our patient was initially diagnosed with Moyamoya disease after presenting with cerebral vasculature complications and went on to develop multiple extracranial complications ultimately leading to death. Clinicians should be aware of the cardiovascular complications seen in Moyamoya patients and assess for the potential benefit of aggressive medical management in preventing coronary plaque progression. It is crucial that these patients are counseled on their risk of ischemic heart disease and informed about the optimal timing for cardiovascular medical evaluation.
